# Supplementary figures and images for: Patterns of Gene Family Evolution and Selection Across Daphnia
Source: Ecol Evol. 2025 May 24;15(5):e71453. doi: 10.1002/ece3.71453 (PMC12102780; doi:10.1002/ece3.71453)

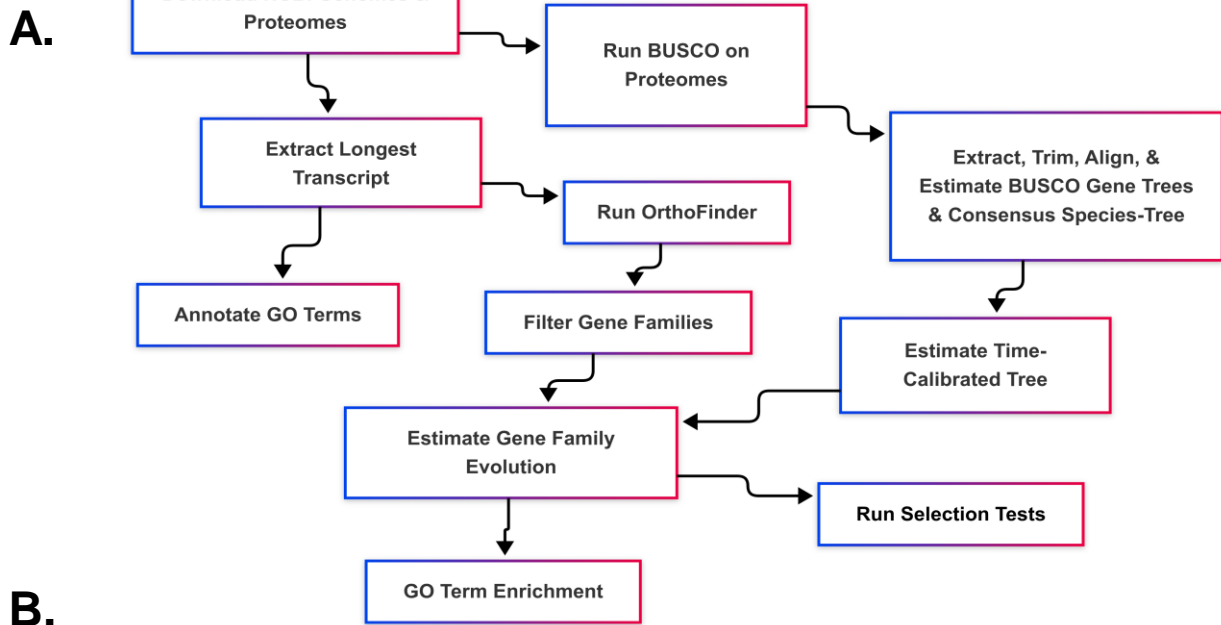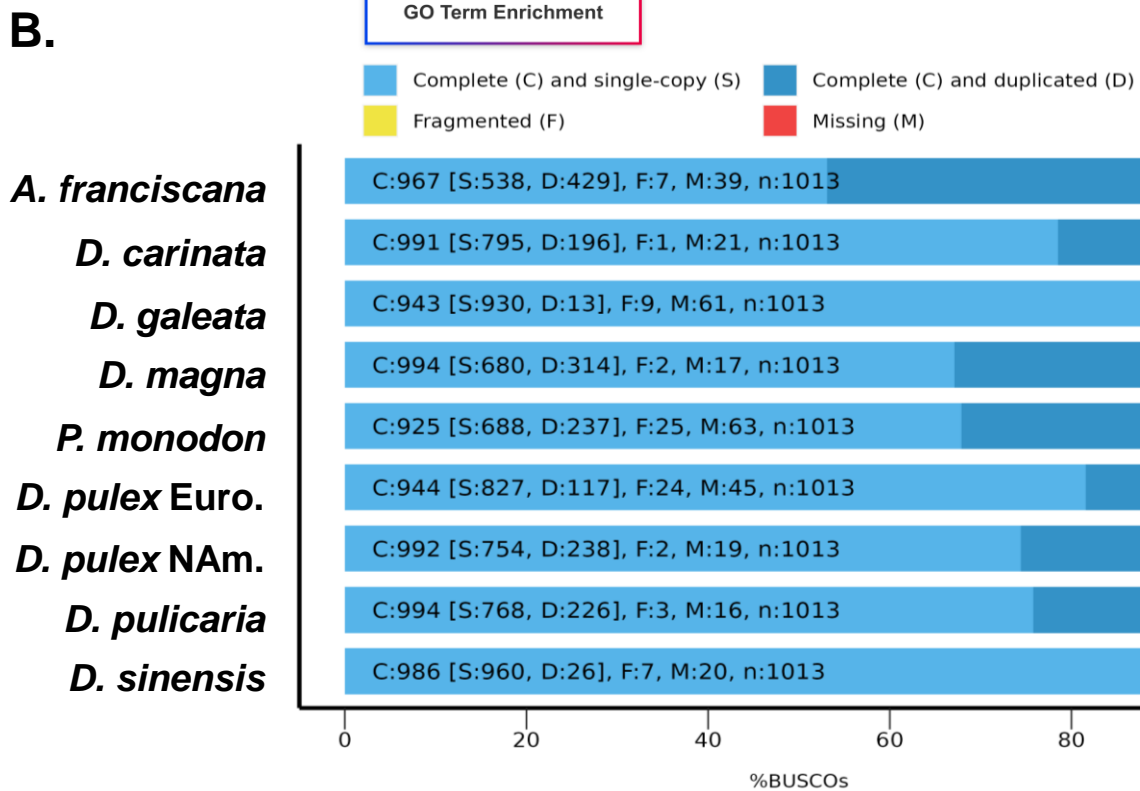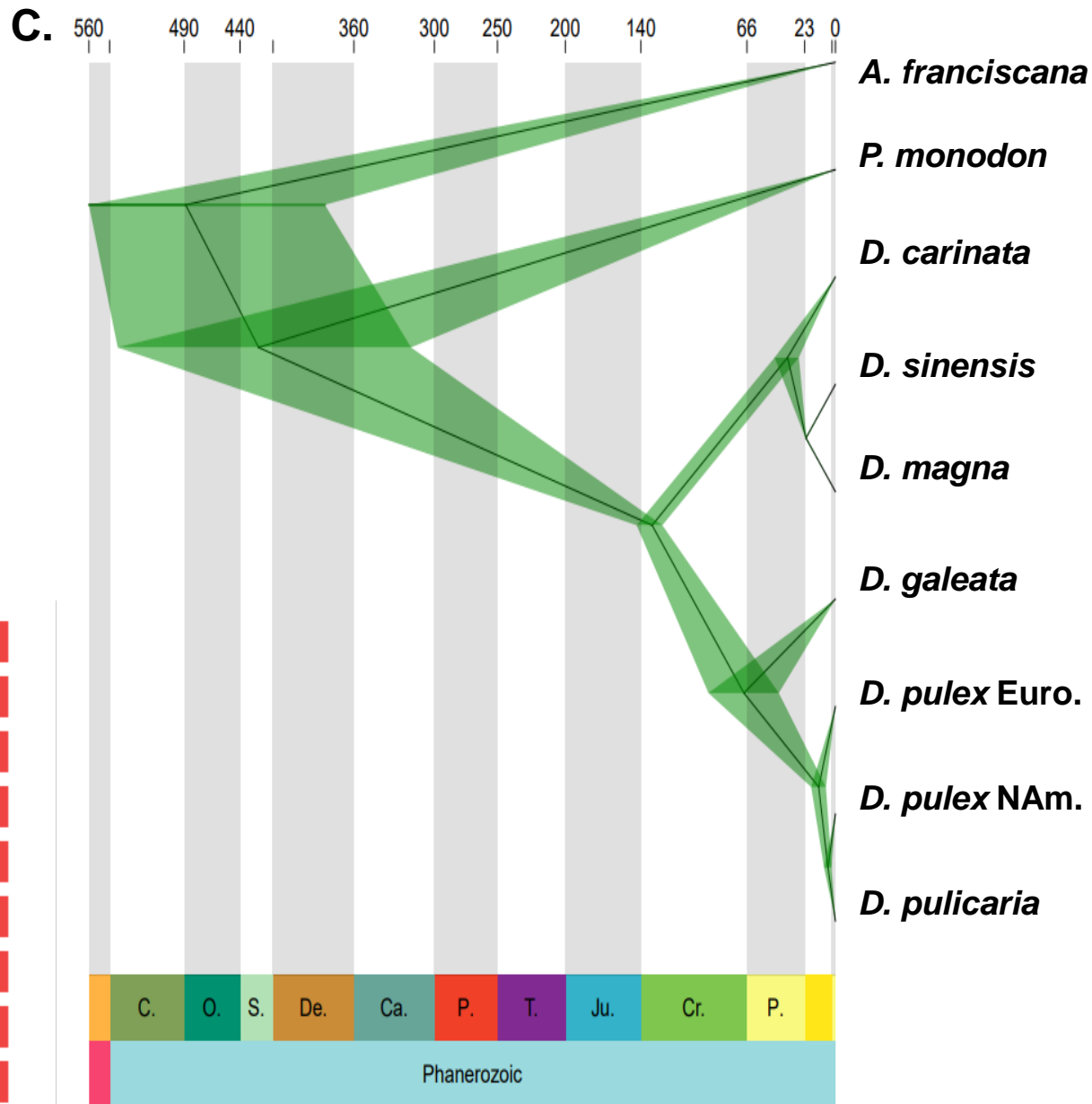

Supplement: Supplementary file 1 — Figure S1. Computational biology pipeline and BUSCO species tree. (A) Nextflow pipeline showing the flow of data between processes starting with a list of RefSeq/GenBank identifiers. (B) BUSCO scores denoting the number of genes that are complete, single‐copy, duplicated, fragmented, missing, and the total across each of the genomes against the Arthropoda dataset. (C) Time‐calibrated phylogenetic tree of the whole‐genomes. The tree was built with the BUSCO genes that were complete and present within each genome. We ran each tree for 5 million Markov chain Monte Carlo generations. [file ECE3-15-e71453-s004.pdf]

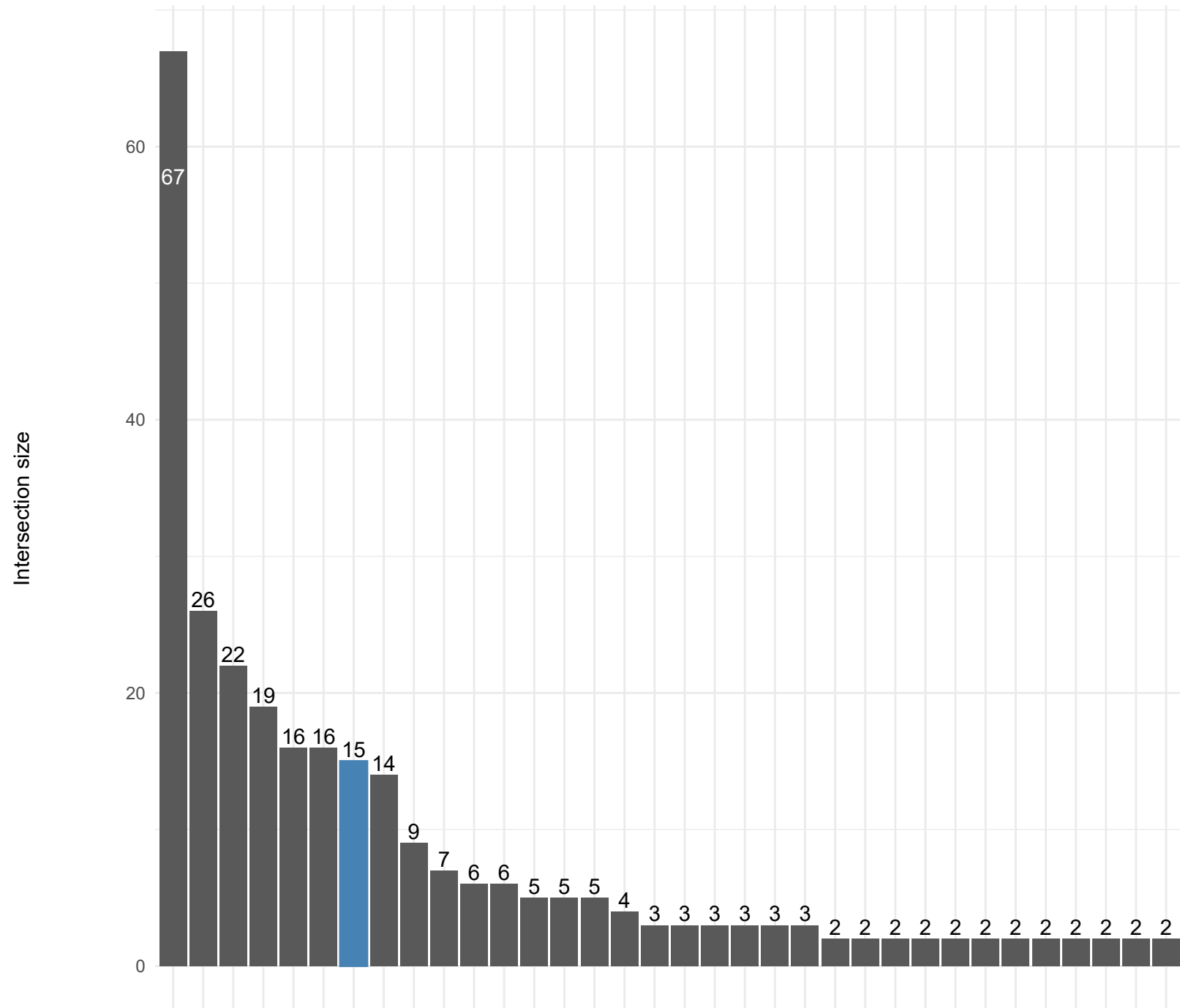

## Expanded GO terms

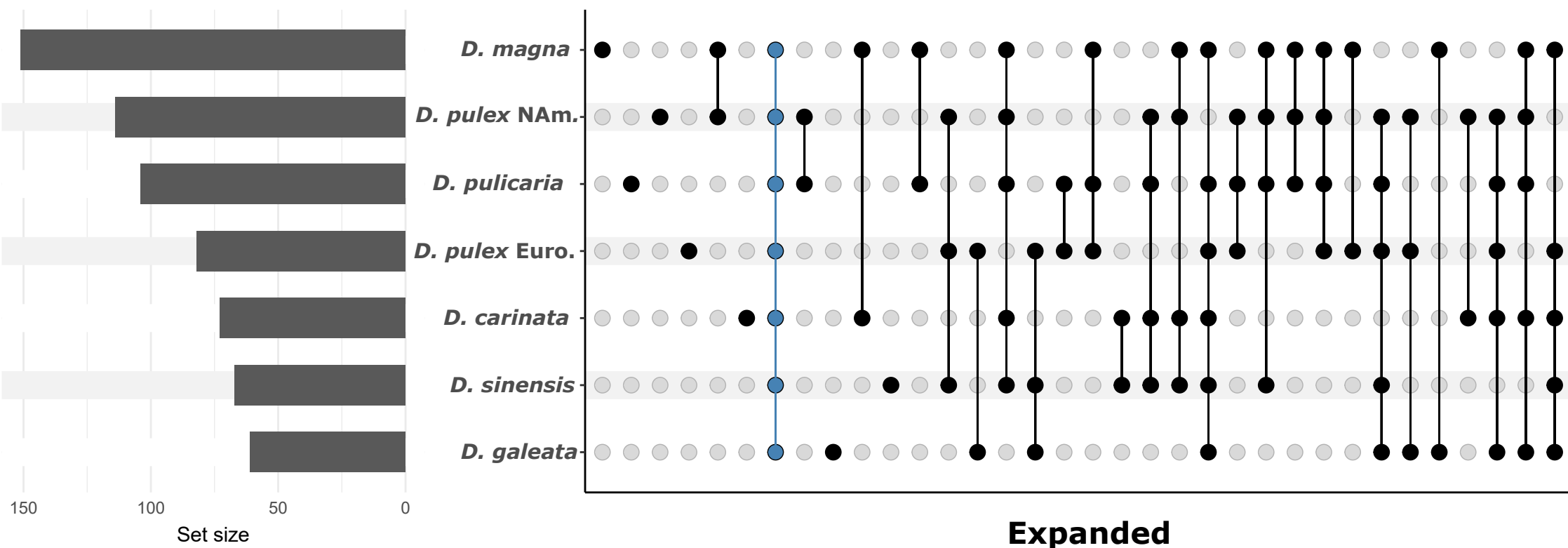

Supplement: Supplementary file 3 — Figure S3. Upset plot of the enriched gene ontology terms for expanding gene families. The blue colored column denotes the GO terms shared by all species. We only show GO term combinations that appear at least twice and also remove outgroup comparisons. The insertion size (y‐axis of the above barplots) is the number of GO terms shared in each group by set combination. Set size (x‐axis of the side barplots) are the number of significant GO terms within each species. These terms are not semantically reduced. [file ECE3-15-e71453-s003.pdf]

## *H2-oxoglutarate and iron-dependent oxygenase JMJD4-like*

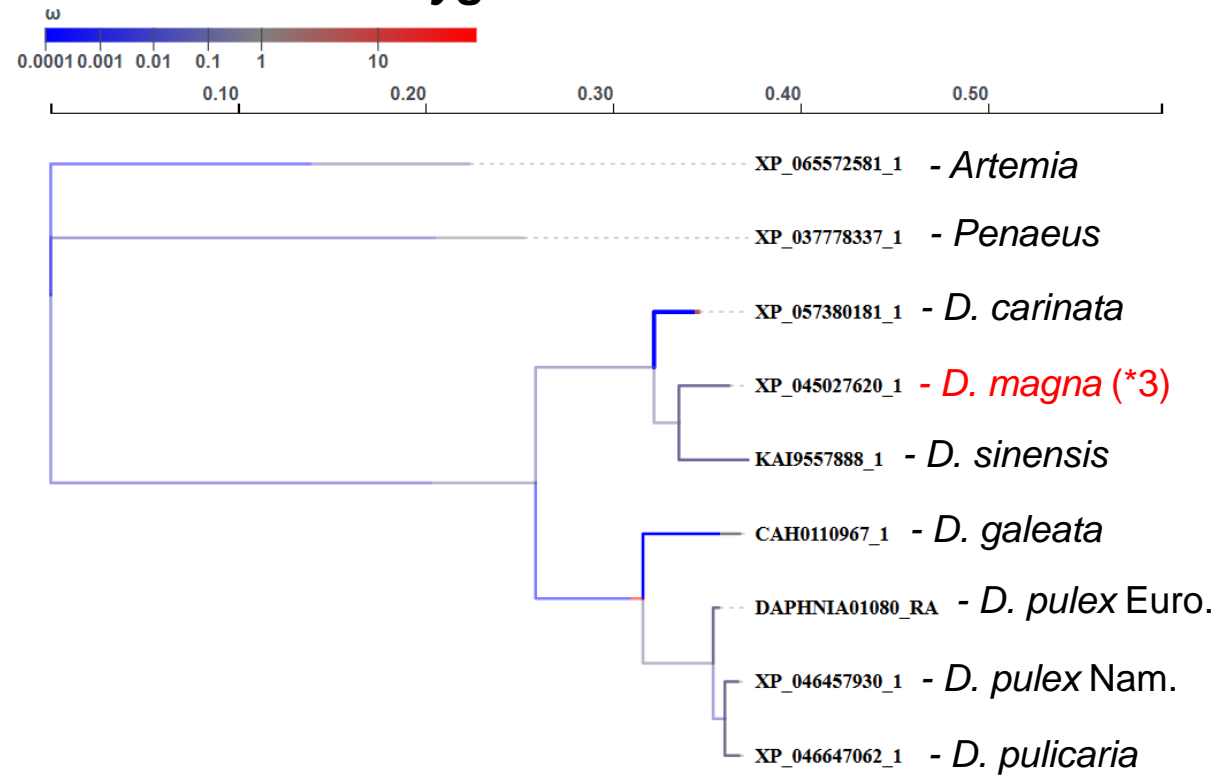

Supplement: Supplementary file 4 — Figure S4. Heme response gene family with expansions, but no positive selection. The thickness of branches indicates the level of significance. If a tip label is colored red, that indicates that a significant expansion occurred with the number of expanded genes in parentheses. The gene names are included as tip labels with the species name. The color of branches indicates the estimated dN/dS. [file ECE3-15-e71453-s001.pdf]

## Glycoprotein-N-acetylgalactosamine 3- beta-galactosyltransferase

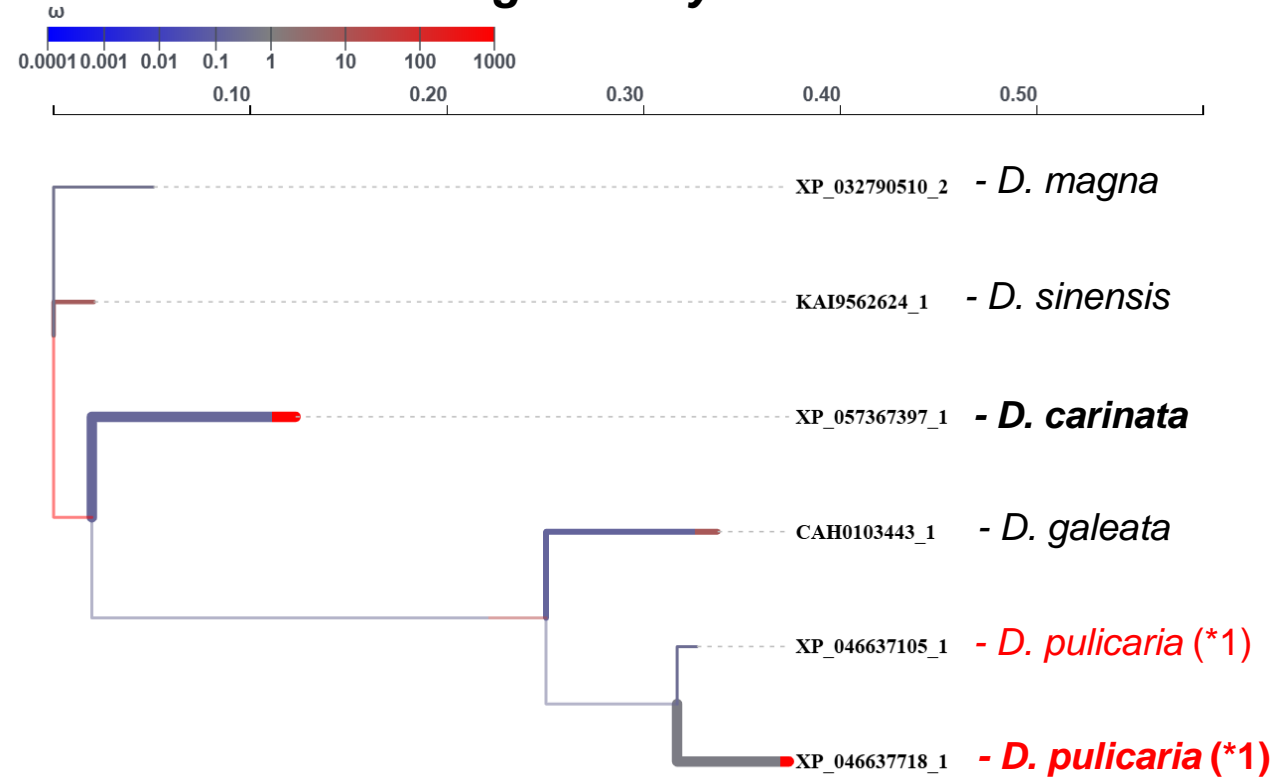

Supplement: Supplementary file 5 — Figure S5. Glycoprotein synthesis gene family undergoing expansions and positive selection. The thickness of branches indicates the level of significance. If a tip label is colored red, that indicates that a significant expansion occurred with the number of expanded genes in parentheses. The gene names are included as tip labels with the species name. The color of branches indicates the estimated dN/dS. [file ECE3-15-e71453-s005.pdf]
