# Supplementary material for: Patterns of Gene Family Evolution and Selection Across Daphnia
Source: Ecol Evol. 2025 May 24;15(5):e71453. doi: 10.1002/ece3.71453 (PMC12102780; doi:10.1002/ece3.71453)
Supplement: Supplementary file 2 — Figure S2. Upset plot of the enriched gene ontology terms for contracted gene families. The blue colored column denotes the GO terms shared by all species. We only show GO term combinations that appear at least twice and also remove outgroup comparisons. The insertion size (y‐axis of the above barplots) is the number of GO terms shared in each group by set combination. Set size (x‐axis of the side barplots) are the number of significant GO terms within each species. These terms are not semantically reduced. [file ECE3-15-e71453-s002.pdf]

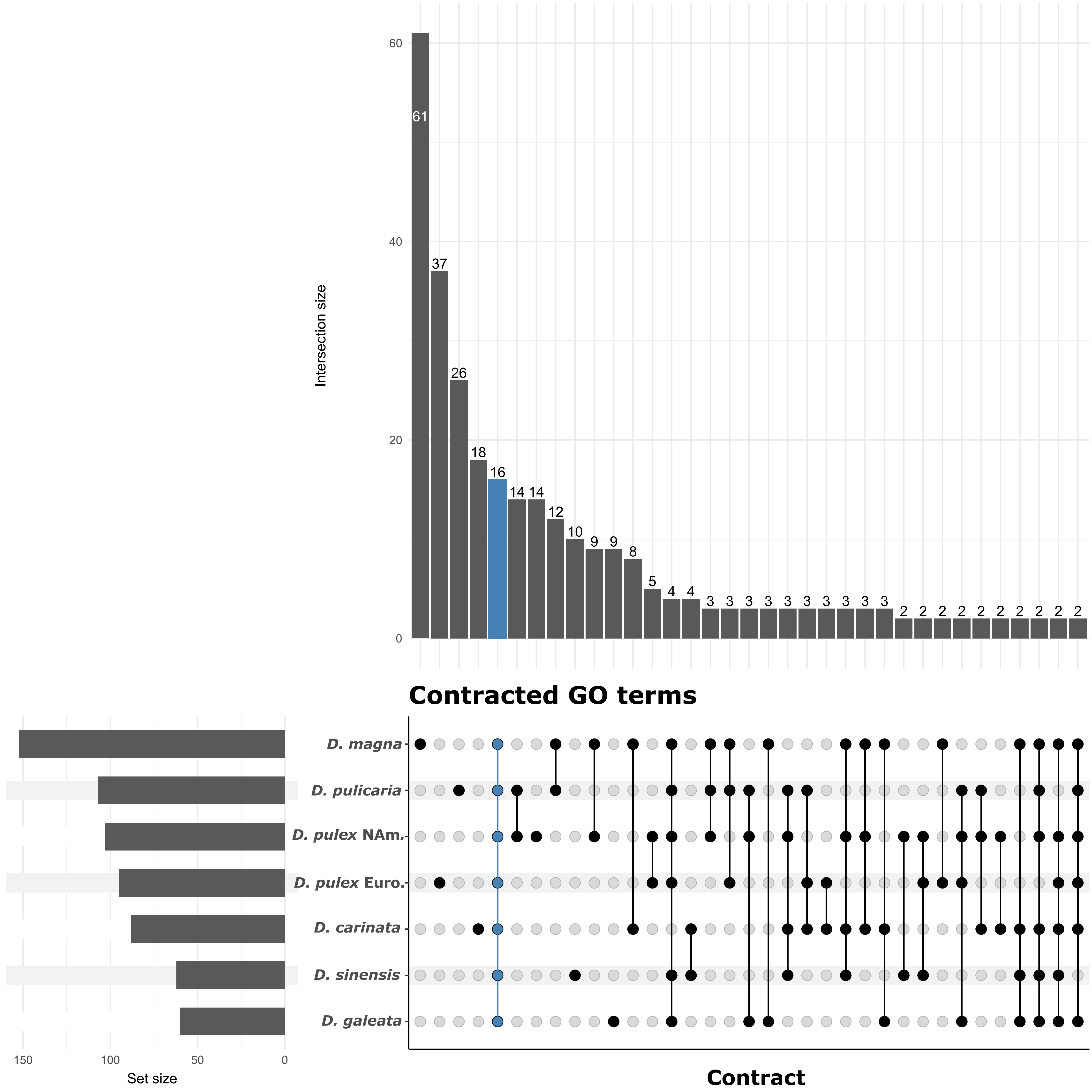

Contracted GO terms
Intersection size
60
40
20
0
61
37
26
18
16
14
14
12
10
9
9
8
5
4
4
3
3
3
3
3
3
3
3
3
3
2
2
2
2
2
2
2
2
2
2
D. magna
D. pulicaria
D. pulex NAm.
D. pulex Euro.
D. carinata
D. sinensis
D. galeata
150
100
50
0
Set size
Contract
